# Cross-cultural adaptation and validation of the rapid assessment of physical activity questionnaire (RAPA) in Hungarian elderly over 50 years

**DOI:** 10.1186/s13102-022-00512-3

**Published:** 2022-07-16

**Authors:** Erika Viktória Miszory, Alexandra Makai, Annamária Pakai, Melinda Járomi

**Affiliations:** 1 Faculty of Health Sciences, Doctoral School, University of Pécs, Pécs, Hungary; 2 Hungarian Defence Forces Medical Centre, Institute of Rehabilitation Hévíz, Hévíz, Hungary; 3 Faculty of Health Sciences, Institute of Physiotherapy and Sport Sciences, University of Pécs, Pécs, Hungary; 4 Faculty of Health Sciences, Institute of Nursing Sciences, Basic Health Sciences and Health Visiting, Department of Health Visiting and Prevention, University of Pécs, Szombathely, Hungary

**Keywords:** Elderly, Hungary, Physical activity, Questionnaire, Validity

## Abstract

**Background:**

Reliable and valid instruments are needed to estimate physical activity levels. The aim was to culturally adapt the “Rapid Assessment of Physical Activity” (RAPA) into Hungarian and to investigate the validity and reliability of this adapted version in the elderly over 50 years.

**Methods:**

In our cross-sectional study 222 subjects were recruited in Hungary between December 2020 and January 2021(age 61.1 ± 7.9 years, 28% male). Criterion validity of RAPA and International Physical Activity Questionnaire (IPAQ)—Hungarian long version was tested by Spearman’s rank correlation. The examination of repeatability was based on a group of 32 people, and on the one-week test–retest reliability approach, and in addition to this during the statistical analysis intra-class correlation coefficient was calculated. To examine the sensitivity and specificity of the RAPA, negative and positive physical activity values were calculated from the results of the long version of the IPAQ and the RAPA. We tested 4 hypotheses (3 validity, 1 reliability). We considered acceptable validity and reliability if > 75% of hypotheses were confirmed. *Results:* All of the hypotheses (100%) were confirmed. Based on results of the validity testing of the newly adapted questionnaire was showed a moderate correlation between the examined measurement tools (R = 0.542, *p* < 0.001). The test–retest results of the questionnaire (N = 32, R = 0.988, *p* < 0.001) showed strong association.

**Conclusion:**

RAPA showed fair to moderate validity and strong test–retest reliability similar to other studies. Based on our study’s results the RAPA is a valid and reliable questionnaire to measure the elderly Hungarian population’s physical activity.

**Supplementary Information:**

The online version contains supplementary material available at 10.1186/s13102-022-00512-3.

## Background

Aging is a multifactorial and irreversible process associated with a significant decrease in muscle mass and neuromuscular function. One of the most effective ways to counteract this is exercise [1]. According to 2020 data, 9% of the world's population is over 65, compared to 19% in Europe. By 2050, the elderly population in more developed countries is projected to exceed that of young people [2]. According to a 2019 study, the prevalence of physical inactivity increases significantly with age: one in five people in the world is physically inactive, and one-third of adults and four-fifths of young people are less than the recommended level of physical activity (PA) [3]. In more developed countries it causes the deaths of 5 million people worldwide [4]. According to the World Health Organization’s (WHO) definition, people between the ages of 5 and 17 should spend at least 60 min a day on moderate-intensity or intense exercise. For the 18- to 64-year-old age group, the minimum recommended activity per week is 150–300 min of moderate-intensity or 75–100 min of vigorous—intense exercise (possibly a combination of the two), and those above 65 years of age are recommended to maintain health [5]. The detrimental health consequences of physical inactivity are important from both a clinical and research perspective. Risk identification and initiatives to change PA all require clinicians and researchers to have a clear understanding of the assessment of PA. By PA we mean all energy-related exercise. This includes any movement that is necessary for leisure, to get to and from places, or is part of one’s work (for example walking, cycling, sports, active recreation, and play). We consider someone to be active when they reach a level of physical activity appropriate to their age. When an individual does not meet the recommended level of PA, he or she will be classified as physically inactive. PA and sedentary behaviors are not the opposite of each other, as it is possible that those who spend most of their time sitting can reach the age level of PA in their free time [6]. By sedentary lifestyle is meant sitting, reclining, or lying posture associated with an energy expenditure ≤ 1.5 METs [7]. There are several subjective [8] and objective measurement [9] methods for evaluating PA. Subjective evaluations include diaries and questionnaire surveys. Surveys continue to play a key role, according to a guide published in 2013 [10]. Its advantages include cost-effectiveness, ease of use, and measurement accuracy of intensive activity, and the definition of discrete categories of activity levels (e.g. low, medium, high) [11, 12]. Overall, questionnaire validation studies show a strong correlation with the criteria for intense PA, but are generally less accurate for mild to moderate intensity activities [11]. The popularity of the questionnaire survey among the elderly is also indicated by the fact that the surveys among the over 55 years have specific methodological considerations, which typically relate to physiological and psychological factors related to age-related cognitive function and deterioration in health [13]. Although there are two international, validated questionnaires in Hungary to assess PA [14, 15], none of them focus specifically on the older age group. The Rapid Assessment of Physical Activity Questionnaire (RAPA) is a self-assessment tool that has been shown to be a good tool for measuring PA in a clinical setting [16]. Its advantage is that it uses images to represent different PAs to make it easier for users to understand, uses little time (about two minutes), and can also be used as a health education tool [17]. Another benefit is that RAPA assesses strengthening and stretching training habits. As recommended by the WHO, adults should also do muscle-strengthening activities at moderate or greater intensity that involve all major muscle groups (on 2 or more days a week) [5], since they are prospectively associated with decreased risk of incident functional limitation in generally healthy, middle-aged and older adults [18]. RAPA is a valid measure of PA that has been used in several clinical trials over the past decade. However, there are concerns about the test–retest, which was not examined in the original validation study [16]. The aim of our research is to culturally adapt the Hungarian version of the questionnaire (RAPA), to investigate the validity and reliability of this adapted version, and to assess the PA of (a) the retired persons (b) the active workers, (c) the persons regularly doing sports up to the age of 30 and (d) the persons not doing sports up to the age of 30.

## Methods

### Participants

Our cross-sectional study was conducted in Hungary between December 2020 and January 2021. Participants were included in our research through online recruitment, and our target group was based on a non-random, convenience sample. Only self mobilized participants aged older than 50 years were included in the study.

To determine the minimum sample size, we used a multiplier of ten to the number of items in the questionnaire as recommended by studies aiming to achieve adequate reliability [19]. To increase the strength of the analysis, we increased this sample size further. Dementia patients, users of assistive devices that greatly affect PA (wheelchair lifestyle and limb prosthesis) and non-Hungarian native speakers, who couldn’t speak and understand Hungarian, read, and write were excluded from the research. The aim of the study was explained to each participant and written informed concurrence was attained before the research began by trained experimenters. The research ethics license was issued by the Regional Research Ethics Committee of Markusovszky Hospital in Szombathely (Hungary, Vas county), license number: 28/2020.

During data collection, two questionnaires got completed by the participants, the Hungarian version of the International Physical Activity Questionnaire (IPAQ) [15] and the Rapid Assessment of Physical Activity (RAPA) [20] (Additional file 1: RAPA_English version). A subsample of 32 people was asked to complete the two questionnaires again, seven days after the first assessment.

### Translation and cross-cultural adaptation

The translation of the RAPA questionnaire into Hungarian was done in consultation with the original author, while its validation was carried out according to the guidelines formulated by Beaton et al. [21], which included the followings: translation, synthesis, reversal, pre-testing, internal consistency testing, external validation with another questionnaire, and test–retest analysis.

As part of the translation process, the English-language questionnaire was first translated by a translator with no medical knowledge and then by a physiotherapist. Comparing the results, a synthesis was created, which was translated back into English by an independent translator. For the final version, we performed international harmonization with the available multilingual versions. The final questionnaire (Additional file 2: RAPA_Hungarian version) was the pre-tested on a group of 32 people over the age of 50.

### Validity and reliability

Originally, the questionnaire was compared with the “Community Healthy Activities Model Program for Seniors” (CHAMPS) [22] questionnaires, which is not yet available in Hungary, so for the validation, we chose the IPAQ, which is applicable to support the external validation process [15]. We assessed construct validity by testing the hypothesized theoretical relationships between the RAPA and the comparison measures. We hyphothesed, that (H1) the correlation between RAPA scores and the IPAQ questionnaire scores will be greater than 0.50. and greater than the correlation between the RAPA scores and age (H2) and Body Mass Index (BMI) (H3), conducted by a Spearman’s rank correlation analyses (ρ).

### Instruments

#### Rapid Assessment of Physical Activity (RAPA)

In 2006, Topolski et al. [16] at the University of Washington, USA, developed the RAPA questionnaire for rapid assessment of PA in the population over 50, thus, to enable the easy use and interpretation of the data as well. It got developed by reviewing and evaluating existing questionnaires that proved to be not only reliable but also valid in comparison to the longer CHAMPS questionnaires [22].

The questions in the nine-item questionnaire range from a sedentary lifestyle to regular and strenuous PA, and from strength training to flexibility-enhancing exercises [16]. The instructions for completing the questionnaire are based on a brief description of the three levels of PA (light, medium, and intense), with graphical and textual representations to help judge the type of activities in each category. It takes maximum of 5 min to complete it. It has nine items that have 2 response options: yes/no. Each “yes” answer is evaluated with 1 point, so the total score of the first seven elements can be a maximum of 7 points. We do not consider the result below 6 points to be optimal. The score obtained by the respondent can be classified into one of the following five levels of PA: (1) sedentary lifestyle; (2) less active; (3) performing regular, light activity; (4) less but regularly active; (5) regularly active. The first 7 questions measure the level and intensity of the PA during free time (RAPA 1), on the other hand, the last two questions include strength training and flexibility items (RAPA 2), which must be scored separately: strength training = 1, flexibility = 2, or both = 3, neither = 0 points. Another advantage of the questionnaire is that it is easy to understand through its sixth-grade level wording, so it can also be completed even by people with cognitive impairments.

#### International physical activity questionnaire long version (IPAQ)

We used the Hungarian long version of the IPAQ to simultaneously test the validity of the RAPA. The questionnaire contains 27 items asking about the frequency, duration (minutes/week), and intensity of activities in the last 7 days. The items examined as part of the IPAQ form were work, transportation, housekeeping, leisure activities, and time spent sitting related. The results were obtained based on the scoring protocol of the questionnaire [23, 24] and in addition to that, the duration of each activity was summarized as well.

### Statistical analysis

Descriptive and mathematical statistics were used in the series of statistical calculations. Data were entered into Microsoft Excel and analyzed with the help of IBM SPSS 27.0 (IBM Corporation, Armonk, NY, USA). To present quantitative data, we calculated the mean and the standard deviation (SD). To assess the construct validity, we hypothesized that there would be a positive significant correlation between the RAPA (RAPA1 and RAPA2) and IPAQ. Additionally, we hypothesized that the RAPA would be negatively correlated with age and BMI. We tested these hypotheses by Spearman’s rank correlation coefficients. The validity was considered as acceptable if the ρ was more than 0.3. The examination of repeatability was based on a randomly selected group of 32 people, and one-week test–retest reliability approach, and intra-class correlation coefficient (ICC) was calculated during statistical analysis. We considered an ICC of ≤ 0.5 as poor, 0.51- 0.74 as moderate, 0.75–0.89 as good, and ≥ 0.90 as excellent reliability. We hypothesized (H4), that the reliability of the RAPA will be greater than 0.90. To examine the sensitivity and specificity of the RAPA questionnaire, negative and positive PA values were calculated from the results of the IPAQ and RAPA questionnaires. RAPA total score above 5 and moderate-intense activity rate above 150 min received a positive evaluation. Our results were considered significant at *p* < 0.05.

## Results

### Characteristics of the participants

222 people were involved in the research (159 women and 63 men), with a mean age of 61.1 ± 7.9 years (min–max: 50–90). 51.4% of respondents are still active workers and less than half (48.6%) are retired. 87.4% of the participants (194 people) indicated that they already have some form of illness. We found a significant correlation between neurological diseases (*p* = 0.032) and testicular and prostate problems (*p* = 0.044) between the individual disease types and the total RAPA score. The characteristics of the individuals involved in the research are presented in Table 1.

**Table 1:** Characteristics of the participants (N = 222)

	Mean	SD^†^
Age (years)	61.1	7.9
Weight (kg)	77.7	16.9
Height (cm)	166.3	8.6
BMI^‡^ (kg/m^2^)	27.9	5.2

		**N**	**%**
Sex	Female/male	159/63	71.6/28.4
Vocational cituation	Active worker	114	51.4
	Pensioner	108	48.6
Physical nature of the occupation of active workers (N = 114)	Physical work	13	11.4
	Sitting work	80	70.2
	Mixed	21	18.4
Fall in the last one year	Yes/no	63/159	28.4/71.6
Use of walking aids	Yes/no	23/199	10.3/89.7
Doing sports (at least twice a week)	Yes/no	84/138	37.8/62.2
Diseases	Yes/no	194/28	87.4/12.6
Allergy, asthma		36	16.2
Metabolic disease		12	5.4
Autoimmun disease		11	4.9
Skin disease		13	5.8
Cancer		15	6.7
Gastrointestinal disease		19	8.5
Testis, prostate problems		2	0.9
Hypertonia		110	49.5
Visual impairment		59	26.6
Neurological disease		15	6.7
Gynecological disease		4	1.8
Orthopaedic disease		44	19.8
Psychiatric disease		3	1.3
Rheumatological disease		60	27
Heart,- vascular disease		23	10.4
Kidney,- urinary problems		10	4.5

^†^Standard deviation

^‡^Body mass index

### Reliability testing of the RAPA

The RAPA was calculated as 0.996 (95% CI 0.992–0.998), and interpreted as having very good test–retest reliability. The hypothesis for reliability was confirmed. The coefficients also exceed 0.6 for each of the nine items. (Table 2). We found a strong correlation between the results of the two measurements (*p* < 0.001).

**Table 2:** Test–retest reliability of the Rapid Assessment of Physical Health using Intraclass Correlation Coefficients (N = 32)

RAPA* items	Intraclass Correlation	95% CI		*p*
		Lower	Upper	
„I rarely or never do any physical activities.”	0.844	0.680	0.924	< 0.001
„I do some light or moderate physical activities, but not every week.”	0.686	0.358	0.847	< 0.001
„I do some light physical activity every week.”	1.000	1.000	1.000	
„I do moderate physical activities every week, but less than 30 min a day or 5 days a week.”	0.936	0.870	0.969	< 0.001
„I do vigorous physical activities every week, but less than 20 min a day or 3 days a week.”	0.921	0.839	0.962	< 0.001
„I do 30 min or more a day of moderate physical activities, 5 or more days a week.”	0.922	0.839	0.962	< 0.001
„I do 20 min or more a day of vigorous physical activities, 3 or more days a week.”	0.916	0.827	0.959	< 0.001
„I do activities to increase muscle strength, such as lifting weights or calisthenics, once a week or more.”	1.000	1.000	1.000	
„I do activities to improve flexibility, such as stretching or yoga, once a week or more.”	1.000	1.000	1.000	
RAPA total	0.996	0.992	0.998	< 0.001
RAPA part 2	1.000	1.000	1.000	

^*^RAPA Rapid Assessment of Physical Health Questionnaire

### Criterion validity of the RAPA

The two questionnaires showed notable connection with a medium correlation (R = 0.542, *p* < 0.001). On the other hand, the second half of the RAPA, which includes strength training and flexibility items, assumes a definite but weak relationship (R = 0.251, *p* < 0.001), so the higher the IPAQ value, the higher the score, the PA of the participants should also be evaluated based on the RAPA questionnaire. A negative but weak correlation was found between RAPA2 and age (R = − 0.182, *p* < 0.001) and BMI (R = − 0.305, *p* < 0.001). The R values showed that the convergent and discriminate validity (together with construct validity) were acceptable. All the 3 hypotheses (3/3, 100%) for validity were confirmed.

### Sensitivity, specificity, and predictive values

RAPA showed good sensitivity and a positive predictive value. The sensitivity and specificity of RAPA were 84.71% and 56.25%, respectively. The positive predictive value is 82.61% and the negative predictive value is 60.00%.

### Physical activity levels of the participants

The mean total score of the RAPA questionnaire was 5.51 ± 1.55 (min: 1, max: 7). the mean value of the second half of the questionnaire was 1.15 ± 1.25 (min: 0, max: 3). More than 50.5% (112 people) of the respondents do not do any strength or flexibility training, and only 21.2% (47 people) do both activities regularly. The highest proportions of retirees (57.4%) and group of no sport until the age of 30 (61.7%) are those who do not currently perform any strengthening or flexibility exercises. These activities are mostly carried out by the group of sport until the age of 30 (28.7%) and active workers (24.6%). The IPAQ weekly minutes score was 812.34 (SD 1139.11). There was a minimal difference in PA during free time (RAPA1) between retired and active workers, while there was a significant difference between the RAPA2 score, which included strengthening and flexibility exercises. Retirees, like the no regular sport group until the age of 30, currently exercise a small proportion.

51.8% of the sample played sports regularly until they were 30 years old. Most of them (60%) still have regular strength training (8.6%), flexibility (43.4%), or both (48%) training types, so the RAPA2 score is the highest in this group. Despite the fact that participants who did not play sports regularly in their youth still have a high rate of active training (61.7%), they achieved a minimally better score in the RAPA1 score in everyday activities compared to members of the regular sports group up to the age of 30 (Table 3).

**Table 3:** Physical activity patterns of the different subgroups of the sample

Group			RAPA 1*	RAPA 2**	IPAQ^ß^ total weekly minutes
	*N*	Avarage age (min–max)	Mean (SD)		
Retired group	108	66.7 ± 6.5 (50–90)	5.48 (1.53)	0.99 (1.23)	720.9 (929.7)
Actice workers	114	55.8 ± 5.7 (50–74)	5.54 (1.57)	1.29 (1.26)	898.9 (1305.3)
Regular sport until the age of 30	115	60.3 ± 7 (50–77)	5.08 (1.48)	1.44 (1.28)	839.7 (1044)
No regular sport until the age of 30	107	62 ± 8.9 (50–90)	5.19 (1.55)	0.84 (1.15)	782.9 (1237.5)

^*^ Rapid assessment of physical health part 1

^**^Rapid assessment of physical health part 2

^ß^ International physical activity questionnaire long version

## Discussion

Our study has suggested that the Hungarian version of the RAPA was a valid and reliable outcome measure for assessing the level of PA in adults aged older than 50 years. Our 4 predefined hypotheses to assess the validity and reliability were confirmed. The significant positive correlation between the RAPA and IPAQ, and the significant negative (weak) correlation between the RAPA and age, and significant negative (stronger than average) between the RAPA and BMI showed that the convergent and discriminant validity of the RAPA was acceptable.

According to the data obtained by the measuring instrument, 65.8% of the subjects over the age of 50 can be classified as active PA, i.e. they perform 30 min or more of moderate PA 5 or more times a week, or 20 min of strenuous PA 3 or for several days. However, only 21.2% of the respondents do strengthening or flexibility training. The latter is in line with the 2017 Eurobarometer data, according to which only 33% of the Hungarian population do sports regularly [25]. Based on our results, it can be seen that regular sports activities in youth have a positive effect on the regular performance of adult strength and flexibility training exercises. Numerous studies indicate a decrease in PA with advancing age [26, 27], which in our study was mainly reflected in the high proportion (57.4%) of retirees avoiding training (strength training and flexibility training). Based on the results of RAPA1, there was no significant difference in PA during free time between the groups, despite the fact that 70.2% of active workers do sedentary work. This can be explained by the fact that after work, they are likely to perform as much physical activity as recommended.

RAPA has demonstrated construct validity in middle-aged [28, 29] and older adults [16]. RAPA showed a positive correlation with PA level on the PA surveys from the CHAMPS(r = 0.48; *p* < 0.001), the Patient-centered Assessment and Counseling for Exercise (r = 0.56; *p* < 0.001), and the Behavioral Risk Factor Surveillance System (r = 0.59; *p* < 0.001). RAPA has been compared with an (ActiGraph) accelerometer (r = 0.45) [29], but the results showed modest validity compared with this PA measuring methods. A Turkish study found significant positive correlation between the RAPA, PASE (ρ = 0.491, *P* < 0.001) and IPAQ-SF total score (ρ = 0.643, *P* < 0.001), and significant negative correlation between the RAPA and IPAQ-SF sitting time (ρ = − 0.498, *P* < 0.001). These results have shown that the convergent and discriminant validity of the RAPA were acceptable [30].

In a validation study in Chile seeking adults, they were found to be similar, inversely, and significantly linked to BMI (r = − 0,020, *p* < 0.01) [31]. However, a study by Perez et al. examined the relationship between subjects in each RAPA activity level and BMI, where the lower the PA, the higher the BMI (except for the regularly active vs. active category (*p* = 0.83)). The reliability of the instrument was moderate (r = 0.61; K = 0.34).

Among the subjects of the RAPA validation articles, a higher proportion of female participants [29–31] was observed, while at the average age it was much lower (min–max: 18–64) in most cases compared to the subjects of our research [29, 30, 32].

Validation studies for the original English version of these surveys did not report test–retest reliability. The reliability was performed in a study by Vega Lopez et al. [29] (ICC: 0.65, *p* < 0.01) and Cekok et al. [30] (Weighted Kappa: 0.917, 95% CI 0.864–0.969), Test–retest reliability was high when the Hungarian version of the RAPA was administered in duplicate one week apart (ICC = 0.996, *p* < 0.001).

We cannot directly compare the present results with previous validation studies, given the different measurement methods. However, it seems that the Hungarian version was a valid version.

RAPA is also preferred for the assessment of PA in various diseases (Table 4). In a cross-sectional study in Saudi Arabia, in the orthopedic ward of a private hospital in Jeddah, the PA of patients treated with low back pain (LBP) was surveyed based on various demographics such as age, sex, and occupational nature [33]. A total of 318 subjects participated in the study (mean age: 39.82 ± 11.42 years). RAPA scores were significantly correlated with sex, i.e., women had lower PA compared to men. Although obesity is often correlated with low PA [34, 35], surprisingly, researchers have not found an association between these variables. Among the participants 71 were over the age of 50 and based on the RAPA assessment, they were characterized by lower PA in comparison to the subjects of our present research. According to the results of RAPA 2, more than 75% do not engage in any PA to strengthen muscles and promote flexibility.

**Table 4:** Comparison of studies measuring physical activity of patients based on rapid assessment questionnaire

Author (year)	Hermanussen et al. (2016) [37]	Low et al. (2017) [33]	Azfar et al. (2019) [36]	Varghese et al. (2019) [38]	Present research (2022)
N	111	132	362	116	222
Age (year) (mean (SD))	64 ± 10	70.62 ± 7.39	39.82 ± 11.42 > 50 years:71	≤ 60 years:71.55%, > 60 years: 28.45%	61.08 ± 7.9
Target group	One or two hand surgeries over the age of 50	Healthy over 60 years	Low back pain (LBP)	Hemodialysis patients	Over 50 years old
RAPA total score (mean (SD))	6.3 ± 2.6	–	–	2.991 ± 1.198	5.51 ± 1.55

	% (people)				
RAPA 1**					
Sedentary	–	26.5 (35)	47.9 (34)	–	2.3 (5)
Underactive	–	7.6 (10)	38 (27)	–	9.5 (21)
Underactive regular light activities	–	50.8 (67)	9.9 (7)	–	21.6 (48)
Underactive regular	–	3 (4)	1.4 (1)	–	0.9 (2)
Active	–	12.1 (16)	2.8 (2)	–	65.8 (146)
	% (people)				
RAPA 2***					
Activity to improve strength	–	77.3 (102)	16.9 (12)	–	5.4 (12)
Activity to improve flexibility	–	0.8 (1)	4.2 (3)	–	23 (51)
Both (strength and flexibility activity)	–	18.9 (25)	0 (0)	–	21.2 (47)
No Activity to improve muscle strength or flexibility	–	3 (4)	78.9 (56)	–	50.5 (112)

^**^ Rapid assessment of physical health part 1

^***^ Rapid assessment of physical health part 2

In India, the PA of hemodialysis patients was measured using the RAPA questionnaire [36]. Of the 116 patients, 71.55% were under the age of 60 and 28.5% were over 60. Patients had a low mean RAPA score (2,991 ± 1,198). Those with optimal PA (n = 10) had a mean hemoglobin of 9,570 ± 1,703 g/dL, while those with suboptimal PA (n = 106) had a mean hemoglobin of 8,621 ± 1,861 g/dL (*P* = 0.123). Low PA was observed among albumin levels below 3.5 g/dl and vegetarian patients, which is likely due to malnutrition, which is very common in India.

The limitation of our research is that in addition to self-assessment questionnaires, the level of PA can be underestimated or overestimated based on subjective judgment, and the non-randomized design and the imbalance of the sample between males and females may also have skewed the results obtained. An additional limitation is that RAPA is designed for use on an individual basis, as a rapid assessment that physicians can use on their patients to encourage them to increase their PA, so for higher items, the values obtained should be accepted with caution.

## Conclusions

The Hungarian RAPA self-administered form showed fair to moderate validity similar to other European studies. Based on the results of the present research, it can be said that the RAPA questionnaire proved to be a well-applied, valid, and reliable measuring tool in the evaluation of the PA of the Hungarian older age group. It is easy and clear completeness is expected to be more successful among the older generation compared to long and complex questionnaires, making its applicability an adequately applicable assessment tool in everyday life. Furthermore, this is the only questionnaire that also assesses strength and resilience, which are closely related to reducing falling and maintaining independence.

## Supplementary Information

**Additional file 1.** RAPA_angol.pdf; English version of the RAPA questionnaire

**Additional file 2.** RAPA_Hungarian version.pdf; Hungarian version of the RAPA questionnaire

## Data Availability

The datasets used generated and analyzed are available from the corresponding author on reasonable request.

## Abbreviations

BMI: Body mass index
CHAMPS: Community healthy activities model program for seniors
ICC: Inter class correlation coefficient
IPAQ short: International physical activity questionnaire short version
IPAQ: International physical activity questionnaire Hungarian long version
LBP: Low back pain
PA: Physical activity
PASE: Physical activity scale for the elderly
RAPA: Rapid assessment of physical activity questionnaire
SD: standard deviation
WHO: World Health Organization
