# Supplementary material for: Cross-cultural adaptation and validation of the rapid assessment of physical activity questionnaire (RAPA) in Hungarian elderly over 50 years
Source: BMC Sports Sci Med Rehabil. 2022 Jul 16;14:131. doi: 10.1186/s13102-022-00512-3 (PMC9288685; doi:10.1186/s13102-022-00512-3)
Supplement: Supplementary file 1 — Additional file 1. RAPA_angol.pdf; English version of the RAPA questionnaire [file 13102_2022_512_MOESM1_ESM.pdf]

How Physically Active Are You?

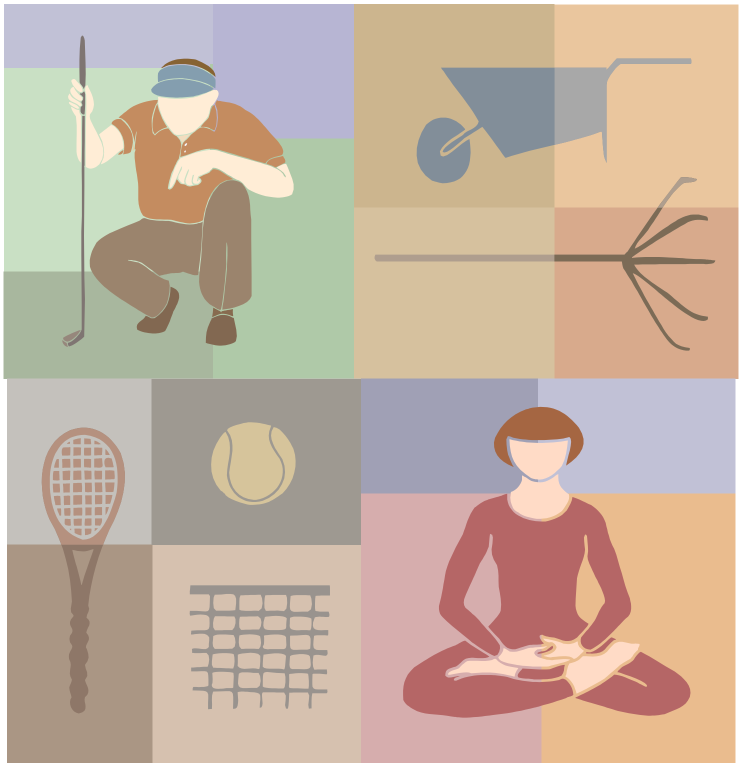

An assessment of level and intensity of physical activity

© 2006 University of Washington Health Promotion Research Center
Do not reproduce without permission, which may be obtained via the Web site:
http://depts.washington.edu/hprc/rapa

Rapid Assessment of Physical Activity

Physical Activities are activities where you move and increase your heart rate above its resting rate, whether you do them for pleasure, work, or transportation.

The following questions ask about the amount and intensity of physical activity you usually do. The intensity of the activity is related to the amount of energy you use to do these activities.

Examples of physical activity intensity levels:

**Light activities**<br>• your heart beats slightly faster than normal<br>• you can talk and sing	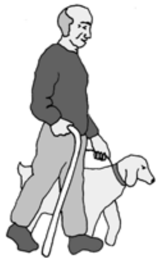 Walking Leisurely 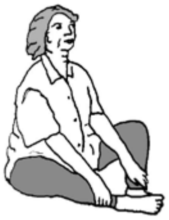 Stretching 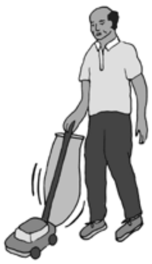 Vacuuming or Light Yard Work
**Moderate activities**<br>• your heart beats faster than normal<br>• you can talk but not sing	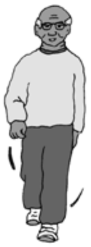 Fast Walking 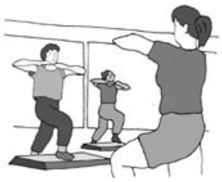 Aerobics Class 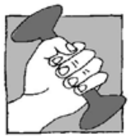 Strength Training 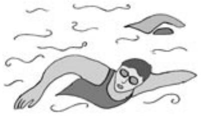 Swimming Gently
**Vigorous activities**<br>• your heart rate increases a lot<br>• you can't talk or your talking is broken up by large breaths	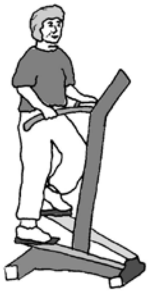 Stair Machine 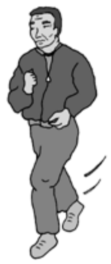 Jogging or Running 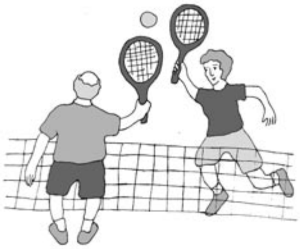 Tennis, Racquetball, Pickleball or Badminton

How physically active are you? *(Check one answer on each line)*

			Does this accurately describe you?	
			**Yes**	**No**
RAPA 1	1	I rarely or never do any physical activities.	☐	☐
	2	I do some **light** or **moderate** physical activities, but not every week.	☐	☐
	3	I do some **light** physical activity every week.	☐	☐
	4	I do **moderate** physical activities every week, but less than 30 minutes a day or 5 days a week.	☐	☐
	5	I do **vigorou**s physical activities every week, but less than 20 minutes a day or 3 days a week.	☐	☐
	6	I do 30 minutes or more a day of **moderate** physical activities, 5 or more days a week.	☐	☐
	7	I do 20 minutes or more a day of **vigorous** physical activities, 3 or more days a week.	☐	☐
RAPA 2 (3 = Both 1 & 2)	1	I do activities to increase muscle **strength**, such as lifting weights or calisthenics, once a week or more.	☐	☐
	2	I do activities to improve **flexibility**, such as stretching or yoga, once a week or more.	☐	☐

ID # ________________________________

Today's Date ________________________

Scoring Instructions

RAPA 1: Aerobic

To score, choose the question with the highest score with an affirmative response. Any number less than 6 is suboptimal.

For scoring or summarizing categorically:

Score as sedentary:

1. I rarely or never do any physical activities.

Score as under-active:

2. I do some light or moderate physical activities, but not every week.

Score as under-active regular – light activities:

3. I do some light physical activity every week.

Score as under-active regular:

4. I do moderate physical activities every week, but less than 30 minutes a day or 5 days a week.
5. I do vigorous physical activities every week, but less than 20 minutes a day or 3 days a week.

Score as active:

6. I do 30 minutes or more a day of moderate physical activities, 5 or more days a week.
7. I do 20 minutes or more a day of vigorous physical activities, 3 or more days a week.

RAPA 2: Strength & Flexibility

I do activities to increase muscle strength, such as lifting weights or calisthenics, once a week or more. (1)

I do activities to improve flexibility, such as stretching or yoga, once a week or more. (2)

Both. (3)

None (0)
