# Supplementary material for: Cross-cultural adaptation and validation of the rapid assessment of physical activity questionnaire (RAPA) in Hungarian elderly over 50 years
Source: BMC Sports Sci Med Rehabil. 2022 Jul 16;14:131. doi: 10.1186/s13102-022-00512-3 (PMC9288685; doi:10.1186/s13102-022-00512-3)
Supplement: Supplementary file 2 — Additional file 2. RAPA_Hungarian version.pdf; Hungarian version of the RAPA questionnaire [file 13102_2022_512_MOESM2_ESM.pdf]

Mennyire aktív fizikailag?

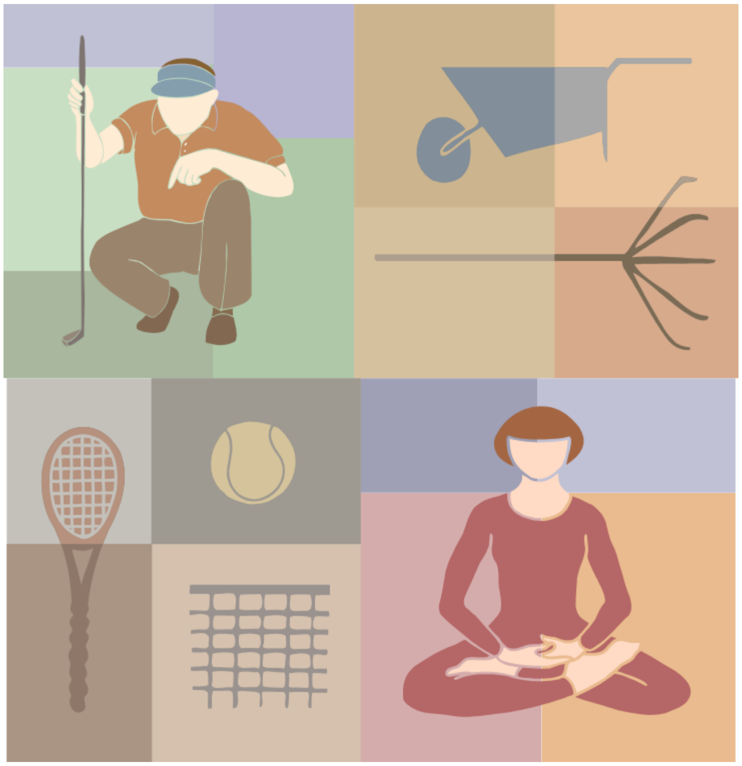

A fizikai aktivitás szintjének és intenzitásának értékelése

© 2006 University of Washington Health Promotion Research Center:
Engedély nélkül tilos másolatot készíteni a dokumentumról. Engedély kérhető az alábbi linkre kattintva:
http://depts.washington.edu/hprc/rapa

A fizikai aktivitás gyors értékelése

A fizikai aktivitások olyan tevékenységek, melyek a mozgással a nyugalmi arány fölé emelik a pulzusszámot, függetlenül attól, hogy szórakozásból, munkavégzésből vagy utazás céljából végzi őket.

A következő kérdések az Ön által általában végzett fizikai aktivitások mértékére és intenzitására vonatkoznak. A tevékenység intenzitása azzal kapcsolatos, hogy Ön mennyi energiát használ ahhoz, hogy ezeket a mozgásokat végrehajtsa.

Példák a fizikai aktivitás intenzitási szintjére:

**Könnyű tevékenységek**<br>• a szíve a normálisnál kissé gyorsabban ver<br>• tud beszélni és énekelni	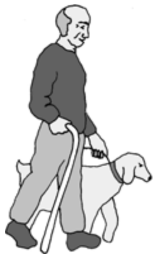 Kényelmes séta<br>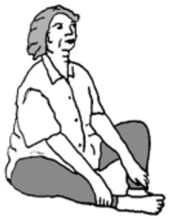 Nyújtás<br>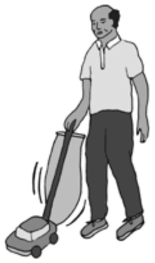 Porszívózás vagy könnyű kerti munka
**Mérsékelt tevékenységek**<br>• a szíve a normálisnál gyorsabban ver<br>• tud beszélni, de nem tud énekelni	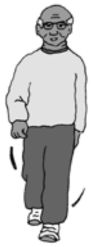 Gyors gyaloglás<br>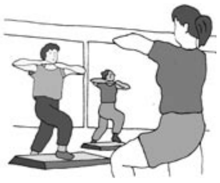 Aerobic óra<br>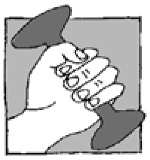 Erőnléti edzés<br>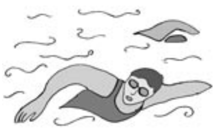 Lassú úszás
**Erőteljes tevékenységek**<br>• a pulzusszám sokat emelkedik<br>• nem tud beszélni, vagy a beszéde darabossá válik és nagy levegőket vesz	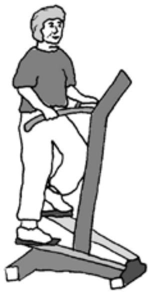 Lépcsőzés<br>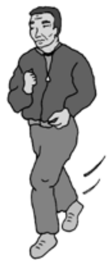 Kocogás vagy futás<br>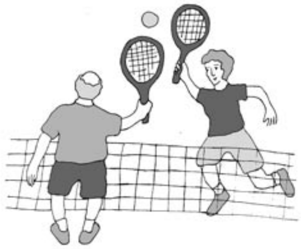 Tenisz, Tollaslabda

Ön mennyire aktív fizikailag? *(minden sorban egy választ jelöljön be)*

Ez pontosan leírja Önt?

			igen	nem
RAPA	1	Ritkán vagy soha nem végzek semmilyen fizikai tevékenységet.	☐	☐
	2	**Kisebb** vagy **közepes** mértékű fizikai tevékenységeket végezek, de nem minden héten	☐	☐
	3	Minden héten végzek **könnyű** fizikai aktivitást	☐	☐
	4	M**érsékelt** fizikai tevékenységet végzek minden héten, de kevesebbet, mint 30 perc / nap, illetve kevesebbet mint 5 nap / hét	☐	☐
	5	**Erőteljes** fizikai tevékenységet minden héten végzek, de kevesebbet, mint 20 perc / nap, illetve kevesebbet mint 3 nap / hét	☐	☐
	6	**Mérsékelt** fizikai tevékenységet végzek minden héten, naponta legalább 30 percet, illetve hetente legalább 5 napot	☐	☐
	7	**Erőteljes** fizikai tevékenységet végzek minden héten, naponta legalább 20 percet, illetve hetente legalább 3 napot	☐	☐
RAPA 2 (3 = Mind az 1 és 2)	1	**Izomerő fejlesztő** gyakorlatokat végzek, mint például súlyemelés vagy saját testsúlyos gyakorlatok, hetente legalább egyszer.	☐	☐
	2	**Rugalmasságot** növelő gyakorlatokat végzek, mint például a nyújtás vagy a jóga, hetente legalább egyszer.	☐	☐

Személy azonosítása:____________________

A mai dátum_____________________________

Pontozási utasítások

RAPA 1: Aerobic

Az értékeléshez válassza ki a legmagasabb pontszámot mutató igaz állítást. A 6-nál kisebb szám nem optimális.

Kategorikus pontozás vagy összegzés:

Pontozza ülő életmódnak:

1. Ritkán vagy soha nem végzek semmilyen fizikai tevékenységet.

Pontozza kevésbé aktívként:

2. Néhány könnyű vagy mérsékelt fizikai tevékenységet végzek, de nem minden héten.

Pontozza kevésbé aktívként, rendszeres - könnyű aktivitást végzőként:

3. Minden héten könnyű testmozgást végezek.

Pontozza kevésbé aktívként, rendszeresen aktívnak:

4. Mérsékelt fizikai tevékenységet végzek minden héten, de kevesebb, mint 30 percet egy nap, vagy 5 napot egy héten.
5. Erőteljes fizikai tevékenységet végzek minden héten, de kevesebb, mint 20 percet egy nap, vagy 3 napot egy héten.

Pontozza aktívként:

6. Mérsékelt fizikai tevékenységet végzek minden héten, naponta legalább 30 percet, illetve hetente legalább 5 napot
7. Erőteljes fizikai tevékenységet végzek minden héten, naponta legalább 20 percet, illetve hetente legalább 3 napot

---

RAPA 2: Erő és rugalmasság

Izomerő növelő gyakorlatokat végzek, mint például súlyemelés vagy saját testsúlyos gyakorlatok, hetente legalább egyszer. (1)

Rugalmasságot növelő gyakorlatokat végzek, mint például a nyújtás vagy a jóga, hetente legalább egyszer. (2)

Mindkettő. (3)

Egyik sem (0)
